# 1117. Case Study. Complications and Challenges in the Treatment of a Burn Patient with a Baclofen Pump

**DOI:** 10.1093/jbcr/irag033.291

**Published:** 2026-04-06

**Authors:** Dasiel Bellido de Luna, Howard G Smith

**Affiliations:** Warden Burn Center at Orlando Health Orlando Regional Medical Center, Ocoee, FL; Warden Burn Center at Orlando Health Orlando Regional Medical Center, Orlando, FL

## Abstract

**Patient Presentation (age range, injury details, relevant history):**

52 y/o male with a past medical history of cerebral palsy, seizures and contracture of the left upper extremity presented with a 12 % total body surface area full thickness burns. The patient had a baclofen pump for treatment of chronic spasticity.

**Clinical Challenges:**

The patient developed Acute Kidney Injury, lethargy and hypothermia. He was readmitted to the ICU for acute metabolic encephalopathy. His creatinine increased to 3.09 with decrease urine output. He was diagnosed with baclofen toxicity. The pump dosage was decreased by 50%. The baclofen pump could not be turned off because it would have required surgical removal and reinsertion of a new pump.

**Management Approach:**

The patient progressed to acute kidney failure secondary to acute tubular necrosis. CRRT was initiated. The baclofen pump dosage was decreased sequentially by 20% every other day, 2 times. Subsequently the patient developed gastroparesis with high nasogastric tube (NGT) output. Baclofen withdrawal was suspected. Baclofen pump dosage was increased progressively by 20% until it reached 80% of home dose.

**Outcomes:**

The patient responded to the decreased baclofen dosage when he demonstrated symptoms of baclofen toxicity. The patient's withdrawal symptoms resolved with the increase in dosage. His renal function recovered and he was discharged home successfully.

**Lessons Learned:**

Major burn patients are at risk of developing metabolic abnormalities and kidney failure. This affects the excretion of chronically administered medications and puts patients at risk for toxicity. Our patient had a baclofen pump. Due to renal failure, he developed toxicity symptomatology. After renal recovery, he developed withdrawal symptomatology. The medical team must remain vigilant for signs of toxicity and withdrawal in patients utilizing these devices and medications.

